# Trends in Empiric Broad-Spectrum Antibiotic Use for Suspected Community-Onset Sepsis in US Hospitals

**DOI:** 10.1001/jamanetworkopen.2024.18923

**Published:** 2024-06-27

**Authors:** Chanu Rhee, Tom Chen, Sameer S. Kadri, Alexander Lawandi, Christina Yek, Morgan Walker, Sarah Warner, David Fram, Huai-Chun Chen, Claire N. Shappell, Laura DelloStritto, Michael Klompas

**Affiliations:** 1 Department of Population Medicine, Harvard Medical School/Harvard Pilgrim Health Care Institute, Boston, Massachusetts; 2 Division of Infectious Diseases, Department of Medicine, Brigham and Women’s Hospital, Boston, Massachusetts; 3 Division of Pulmonary and Critical Care Medicine, Department of Medicine, Brigham and Women’s Hospital, Boston, Massachusetts; 4 Critical Care Medicine Department, National Institutes of Health Clinical Center, Bethesda, Maryland; 5 Critical Care Medicine Branch, National Heart Lung and Blood Institute, Bethesda, Maryland; 6 Division of Infectious Diseases, Department of Medicine, McGill University Health Centre, Quebec, Canada; 7 Commonwealth Informatics, Waltham, Massachusetts

## Abstract

**Importance:**

Little is known about the degree to which suspected sepsis drives broad-spectrum antibiotic use in hospitals, what proportion of antibiotic courses are unnecessarily broad in retrospect, and whether these patterns are changing over time.

**Objective:**

To describe trends in empiric broad-spectrum antibiotic use for suspected community-onset sepsis.

**Design, Setting, and Participants:**

This cross-sectional study used clinical data from adults admitted to 241 US hospitals in the PINC AI Healthcare Database. Eligible participants were aged 18 years or more and were admitted between 2017 and 2021 with suspected community-onset sepsis, defined by a blood culture draw, lactate measurement, and intravenous antibiotic administration on admission.

**Exposures:**

Empiric anti–methicillin-resistant *Staphylococcus aureus* (MRSA) and/or antipseudomonal β-lactam agent use.

**Main Outcomes and Measures:**

Annual rates of empiric anti-MRSA and/or antipseudomonal β-lactam agent use and the proportion that were likely unnecessary in retrospect based on the absence of β-lactam resistant gram-positive or ceftriaxone-resistant gram-negative pathogens from clinical cultures obtained through hospital day 4. Annual trends were calculated using mixed-effects logistic regression models, adjusting for patient and hospital characteristics.

**Results:**

Among 6 272 538 hospitalizations (median [IQR] age, 66 [53-78] years; 443 465 male [49.6%]; 106 095 Black [11.9%], 65 763 Hispanic [7.4%], 653 907 White [73.1%]), 894 724 (14.3%) had suspected community-onset sepsis, of whom 582 585 (65.1%) received either empiric anti-MRSA (379 987 [42.5%]) or antipseudomonal β-lactam therapy (513 811 [57.4%]); 311 213 (34.8%) received both. Patients with suspected community-onset sepsis accounted for 1 573 673 of 3 141 300 (50.1%) of total inpatient anti-MRSA antibiotic days and 2 569 518 of 5 211 745 (49.3%) of total antipseudomonal β-lactam days. Between 2017 and 2021, the proportion of patients with suspected sepsis administered anti-MRSA or antipseudomonal therapy increased from 63.0% (82 731 of 131 275 patients) to 66.7% (101 003 of 151 435 patients) (adjusted OR [aOR] per year, 1.03; 95% CI, 1.03-1.04). However, resistant organisms were isolated in only 65 434 cases (7.3%) (30 617 gram-positive [3.4%], 38 844 gram-negative [4.3%]) and the proportion of patients who had any resistant organism decreased from 9.6% to 7.3% (aOR per year, 0.87; 95% CI, 0.87-0.88). Most patients with suspected sepsis treated with empiric anti-MRSA and/or antipseudomonal therapy had no resistant organisms (527 356 of 582 585 patients [90.5%]); this proportion increased from 88.0% in 2017 to 91.6% in 2021 (aOR per year, 1.12; 95% CI, 1.11-1.13).

**Conclusions and Relevance:**

In this cross-sectional study of adults admitted to 241 US hospitals, empiric broad-spectrum antibiotic use for suspected community-onset sepsis accounted for half of all anti-MRSA or antipseudomonal therapy; the use of these types of antibiotics increased between 2017 and 2021 despite resistant organisms being isolated in less than 10% of patients treated with broad-spectrum agents.

## Key Points

**Question:**

Is broad-spectrum antibiotic use for suspected community-onset sepsis changing over time?

**Findings:**

In this cross-sectional study of 6.3 million adults admitted to 241 US hospitals between 2017 and 2021, half of all anti–methicillin-resistant *Staphylococcus aureus* and antipseudomonal β-lactam antibiotics were prescribed for suspected community-onset sepsis but only 9.5% of suspected community-onset sepsis cases treated with broad-spectrum antibiotics had resistant organisms identified.

**Meaning:**

These results suggest that more attention is needed toward balancing early broad-spectrum antibiotic prescribing for patients with sepsis with limiting overuse for the majority who do not have antibiotic-resistant infections.

## Teaser

This cross-sectional study of US adults hospitalized with suspected community-onset sepsis examines trends in empirical broad-spectrum antibiotic use from 2017 to 2021.

## Introduction

Early administration of appropriate antimicrobial therapy can be life-saving in patients with sepsis and is heavily emphasized by best practice guidelines, quality improvement initiatives, and government metrics.^1,2,3,4,5,6,7,8,9^ The Centers for Medicare & Medicaid Services’ Severe Sepsis and Septic Shock Management Bundle (SEP-1) measure, for example, requires broad-spectrum antibiotic administration within 3 hours of sepsis onset, while the Surviving Sepsis Campaign’s Hour-1 Bundle sets a 1 hour goal.^10,11^

However, many patients initially suspected of sepsis are ultimately diagnosed with noninfectious or viral conditions.^12,13^ Furthermore, most patients who do have bacterial sepsis are infected with pathogens covered by narrower-spectrum antibiotics.^14^ The increasing emphasis on administering immediate antibiotics to all patients with possible sepsis may therefore be driving increased use of unnecessarily broad-spectrum antibiotics, antibiotic-associated adverse events, and antimicrobial resistance without benefitting patients.^14,15,16,17,18^

Several studies examining the period shortly before and after SEP-1 was introduced in 2015 demonstrated significant increases in broad-spectrum and total antibiotic use in US hospitals without corresponding decreases in sepsis-associated mortality.^19,20,21,22^ However, it is unclear to what extent suspected sepsis contributes to overall broad-spectrum antibiotic utilization in hospitals, what proportion of antibiotic administrations turn out to be unnecessarily broad in retrospect, and whether these patterns are changing over time. We therefore analyzed a large cohort of US hospitals to elucidate the epidemiology and trends in empiric broad-spectrum antibiotic use relative to identified pathogens for patients with suspected community-onset sepsis.

## Methods

### Study Design, Population, and Data Source

This was a retrospective cross-sectional study of all adults (aged 18 years or older) admitted to US hospitals in the PINC AI Healthcare Database (Premier Inc), a large database that includes detailed data on approximately 25% of US inpatient admissions from all payers drawn from geographically diverse nonprofit, nongovernmental, community, and teaching hospitals between January 2017 and June 2021.^23^ We focused on the subset of hospitals that reported laboratory and microbiologic data during the study period and met several data quality control checks (eFigure in Supplement 1). Reporting followed the Strengthening the Reporting of Observational Studies in Epidemiology (STROBE) reporting guideline for cross-sectional studies. The study was approved by the institutional review board of Harvard Pilgrim Health Care Institute with a waiver of informed consent because PINC AI is a deidentified dataset.

### Identifying Patients With Suspected Community-Onset Sepsis

We defined *suspected community-onset sepsis* as: (1) blood culture drawn (regardless of result), (2) lactate measurement (regardless of result), and (3) administration of an intravenous antibacterial agent, all on hospital day 0 or 1 (with day 1 being the day of admission; day 0, if applicable, adding time spent in the emergency department the day before admission). We did not use sepsis discharge diagnosis codes because these are variably applied and miss many patients initially treated for sepsis but ultimately diagnosed with other conditions.^24^

Exclusion criteria included (1) missing discharge disposition, (2) transfer from another acute care hospital, subacute health care facility, or hospice, and (3) a discharge diagnosis of COVID-19, because COVID-19 patients were frequently prescribed unnecessary antibiotics and this could distort trends in broad-spectrum antimicrobial utilization.^25^ We also excluded hospitals with fewer than 50 cases of suspected sepsis during the study period.

### Broad-Spectrum Antibiotics of Interest and Definitions of Unnecessarily Broad and Inappropriately Narrow Therapy

We defined broad-spectrum antibiotics as those active against either methicillin-resistant *Staphylococcus aureus* (MRSA) or *Pseudomonas aeruginosa* (eMethods in Supplement 1). Antibiotics were considered unnecessarily broad in retrospect if (1) anti-MRSA therapy was administered but no resistant gram-positive organism was isolated from clinical cultures obtained on hospital days 0 through 4 (MRSA, vancomycin-resistant Enterococcus [VRE], or ceftriaxone-resistant *Streptococcus*; or 2 separate blood cultures with methicillin-resistant coagulase-negative staphylococci from the same or consecutive days), or (2) antipseudomonal β-lactams were administered but no *P aeruginosa* or other ceftriaxone-resistant gram-negative organism was identified in clinical cultures obtained on hospital days 0 through 4. Ceftriaxone resistance was determined based on antimicrobial susceptibility testing (AST) as reported by each hospital. In addition, *Stenotrophomonas maltophilia*, *Achromobacter* species, *Burkholderia* species, and Enterobacterales organisms at high risk for inducible AmpC resistance (*E cloacae*, *K aerogenes*, and *C freundii*) were considered ceftriaxone-resistant regardless of AST testing (eMethods in Supplement 1).^26^ Clinical cultures included any body site; surveillance cultures drawn for infection control purposes were excluded (ie, MRSA nasal swab, VRE rectal swabs). Inappropriately narrow therapy was defined as the absence of anti-MRSA or antipseudomonal β-lactam agents in patients in whom resistant gram-positive or gram-negative organisms were subsequently isolated, respectively.

### Statistical Analysis

We calculated the fraction of all adult inpatients and those with suspected community-onset sepsis who received anti-MRSA and/or antipseudomonal β-lactam agents on hospital days 0 and 1, the proportion that had a resistant gram-positive or gram-negative organism on hospitals days 0 through 4, the proportion of anti-MRSA and/or antipseudomonal therapy that was unnecessarily broad in retrospect, and the proportion of patients who received inappropriately narrow treatment for each calendar year. To assess trends and deviations over the study period (2017-2021), we calculated adjusted odds ratios (aOR) through 2 approaches: first, by considering year as a continuous variable to identify average annual trends; and second, by treating year as a categorical variable with 2017 as the reference. Our analyses employed mixed-effects logistic regression, controlling for both patient-level characteristics (demographics [age, sex, race and/or ethnicity]; comorbidities derived using the Agency for Healthcare Research Quality Elixhauser comorbidity classifier^27^; initial laboratory values [lactate, creatinine, bilirubin, white blood cell count, and platelet count]; need for vasopressors or mechanical ventilation [invasive or noninvasive] on hospital day 2 or prior; and infectious source [determined by *International Statistical Classification of Diseases and Related Health Problems, Tenth Revision (ICD-10)* discharge diagnosis codes flagged as present-on-admission]) and hospital-level characteristics (region, teaching status, urban vs rural, number of beds) as fixed effects. Race and/or ethnicity, as reported by patients and recorded in each hospital’s electronic health record database, was also included given its known association with sepsis treatment patterns and outcomes and was categorized as Asian, Black, Hispanic, White, other (not defined further in the data source), or unknown.^28^ To address the potential correlation of patients within the same hospital and unobserved between-hospital variability, each hospital was included as a random effect in our models.

We conducted 3 sensitivity analyses to examine if trends differed using alternative definitions of suspected community-onset sepsis. First, we used a broader definition that only required a blood culture on hospital day 0 or 1 (with or without lactate measurements and intravenous antibiotics). Second, we used a narrower definition that also required organ dysfunction (based on modified eSOFA criteria^29^) on hospital day 0 or 1 (in addition to blood cultures, lactate measurement, and intravenous antibiotics). Third, we limited the analysis to patients who met the primary definition (blood culture, lactate, intravenous antibiotics) and had positive clinical cultures on days 0 through 4 (excluding common contaminants).

All tests of significance used 2-sided *P* values at <.05. Analyses were conducted using SAS version 9.4 (SAS Institute) and R version 3.3.1 (R Project for Statistical Computing).

## Results

### Study Cohort and Characteristics of Patients With Suspected Community-Onset Sepsis

The final cohort included 6 272 538 adult admissions to 241 hospitals between 2017 and 2021, of whom 894 724 (14.3%) had suspected sepsis on admission (eFigure in Supplement 1). Most hospitals were nonteaching (173 [71.8%]), small (fewer than 200 beds) (125 [51.9%]), urban (169 [70.1%]), and from the South (137 [56.9%]) (eTable 1 in Supplement 1). Among patients with suspected community-onset sepsis, median (IQR) age was 66 (53-78) years (443 465 male [49.6%]; 20 295 Asian [2.3%], 106 095 Black [11.9%], 65 763 Hispanic [7.4%], 653 907 White [73.1%]). Comorbidities were common (eg, 343 458 patients with diabetes [38.4%], 322 040 with chronic lung disease [36.0%]), and 186 545 (20.9%) were admitted to an intensive care unit (ICU) (Table). Patients with suspected sepsis who received empiric MRSA and/or antipseudomonal-β-lactam therapy tended to be more severely ill than those who did not, as indicated by higher rates of vasopressor use, invasive ventilation, lactate levels, ICU admission, and in-hospital death and discharge to hospice (Table).

**Table.** Characteristics of Patients With Suspected Community-Onset Sepsis

Characteristic	Patients with suspected community-onset sepsis, No. (%)		
	Total (n = 894 724)	Receiving empiric anti-MRSA and/or antipseudomonal β-lactams (n = 582 585)	Did not receive empiric anti-MRSA or antipseudomonal β-lactams (n = 312 139)
Age, median (IQR), y	66 (53-78)	65 (52-77)	69 (55-80)
Sex^a^			
Female	451 255 (50.4)	275 168 (47.2)	176 087 (56.4)
Male	443 465 (49.6)	307 415 (52.8)	136 050 (43.6)
Race or ethnicity			
White	653 907 (73.1)	420 237 (72.1)	233 670 (74.9)
Black	106 095 (11.9)	72 670 (12.5)	33 425 (10.7)
Hispanic	65 763 (7.4)	44 332 (7.6)	21 431 (6.9)
Asian	20 295 (2.3)	12 676 (2.2)	7619 (2.4)
Other^b^	41 207 (4.6)	27 422 (4.7)	13 785 (4.4)
Unknown	7457 (0.8)	5248 (0.9)	2209 (0.7)
Comorbidities			
Cancer^c^	106 302 (11.9)	80 447 (13.8)	25 855 (8.3)
Chronic lung disease	322 040 (36.0)	200 123 (34.4)	121 917 (39.1)
Congestive heart failure	256 546 (28.7)	170 827 (29.3)	85 719 (27.5)
Diabetes^d^	343 458 (38.4)	238 595 (41.0)	104 863 (33.6)
Liver disease	94 371 (10.6)	68 127 (11.7)	26 244 (8.4)
Neurologic disease	214 395 (24.0)	151 483 (26.0)	62 912 (20.2)
Kidney disease	245 935 (27.5)	171 392 (29.4)	74 543 (23.9)
AHRQ Elixhauser Mortality Score, median (IQR)	5 (3-8)	5 (3-8)	5 (3-7)
Infectious diagnoses (present-on-admission)			
Pulmonary	316 837 (35.4)	188 398 (32.3)	128 439 (41.2)
Urinary	208 505 (23.3)	125 560 (21.6)	82 945 (26.6)
Intra-abdominal	81 706 (9.1)	60 248 (10.3)	21 458 (6.9)
Skin or soft tissue	133 412 (14.9)	118 219 (20.3)	15 193 (4.9)
Septicemia or bacteremia	343 210 (38.4)	258 905 (44.4)	84 305 (27.0)
Illness severity (days 0-2)			
Vasopressors	74 415 (8.3)	63 156 (10.8)	11 259 (3.6)
Noninvasive ventilation	56 427 (6.3)	35 694 (6.1)	20 733 (6.6)
Invasive ventilation	58 053 (6.5)	46 896 (8.1)	11 157 (3.6)
Lactate 2.0 to <4.0 mmol/L	234 041 (26.2)	159 487 (27.4)	74 554 (23.9)
Lactate ≥4.0 mmol/L	83 483 (9.3)	66 715 (11.5)	16 768 (5.4)
ICU admission	186 545 (20.9)	144 615 (24.8)	41 930 (13.4)
Hospital length of stay, median (IQR), d	4 (2-7)	4 (3-7)	3 (2-5)
Discharge disposition			
Home	603 315 (67.4)	369 577 (63.4)	233 738 (74.9)
Nonacute care facility	177 809 (19.9)	125 681 (21.6)	52 128 (16.7)
Other acute care hospital	2901 (0.3)	2097 (0.4)	804 (0.3)
Left against medical advice	24 033 (2.7)	17 346 (3.0)	6687 (2.1)
Court or law enforcement	1577 (0.2)	1215 (0.2)	362 (0.1)
Hospice	41 953 (4.7)	31 770 (5.5)	10 183 (3.3)
Death	40 124 (4.5)	32 929 (5.6)	7195 (2.3)

Abbreviations: AHRQ, Agency for Healthcare Research Quality; ICU, intensive care unit; MRSA, methicillin-resistant *Staphylococcus aureus*.

SI conversion factors: To convert lactate to milligram per deciliter, divide by 0.111.

^a^ Data on sex were missing or unknown for 4 patients (2 receiving empiric anti-MRSA and/or antipseudomonal β-lactams; 2 not receiving empiric anti-MRSA and/or antipseudomonal β-lactams).

^b^ PINC AI does not list groups included in other.

^c^ Cancer includes lymphoma, solid tumor without metastases, and solid tumor with metastases.

^d^ Diabetes with or without complications.

### Microbiology of Suspected Community-Onset Sepsis

**Figure 1.**
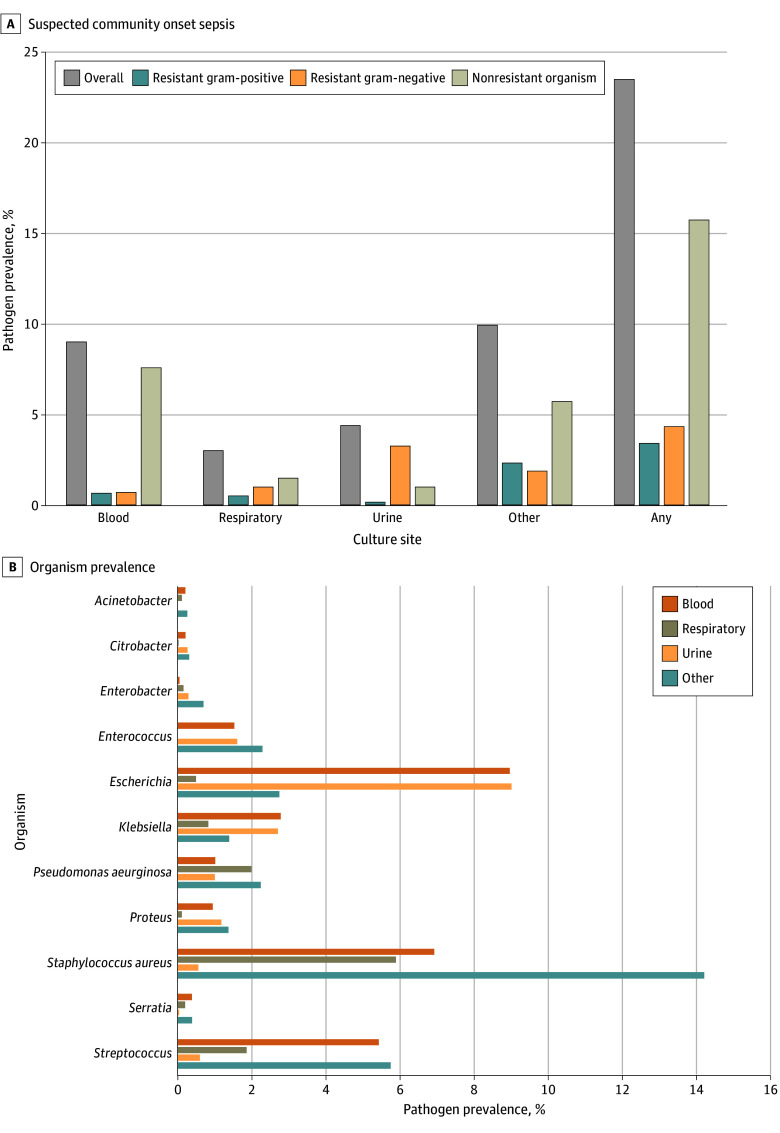
Microbiology of Suspected Community-Onset Sepsis Resistant gram-positive organisms included methicillin-resistant *Staphylococcus aureus* (MRSA), vancomycin-resistant *Enterococcus* (VRE), ceftriaxone-resistant *Streptococcus*, and methicillin-resistant coagulase-negative staphylococci (for blood cultures only). Resistant gram-negative organisms included *P aeruginosa* and any other ceftriaxone-resistant gram-negative pathogen.

Among patients with suspected sepsis, 210 728 (23.6%) had a positive culture on hospital days 0 through 4, including 80 870 (9.0%) with positive blood cultures (Figure 1). Resistant organisms were uncommon: 30 617 (3.4%) had a resistant gram-positive organism (29 205 MRSA [95.4%], 2062 VRE [6.7%], 412 methicillin-resistant Coagulase-negative bacteremia [1.3%], and 192 ceftriaxone-resistant *Streptococcus* [0.6%]); 38 844 (4.3%) had a resistant gram-negative organism (20 216 *P aeruginosa *[52.0%], 5909 ceftriaxone-resistant *E coli* [15.2%], 5319 *Enterobacter* species [13.7%], and 3445 *Klebsiella* species [8.9%]); and 65 434 (7.3%) had either a resistant gram-positive or gram-negative organism.

### Empiric Anti-MRSA and Antipseudomonal β-Lactam Therapy

**Figure 2.**
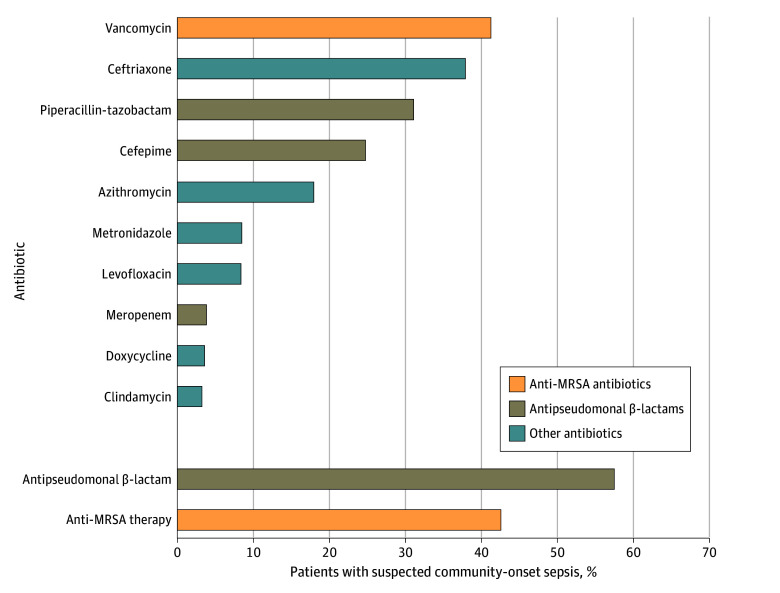
Most Common Empiric Antibiotics Administered to Patients With Suspected Community-Onset Sepsis on Admission MRSA indicates methicillin-resistant *Staphylococcus aureus*.

Among patients with suspected sepsis, 379 987 of 894 724 (42.5%) received empiric anti-MRSA agents on admission, 513 811 (57.4%) received empiric antipseudomonal β-lactam therapy, 582 585 (65.1%) received either empiric anti-MRSA or antipseudomonal β-lactam therapy, and 311 213 (34.8%) received both. The most common empiric antibiotic was vancomycin (41.2%), followed by ceftriaxone (37.8%), piperacillin-tazobactam (31.1%), and cefepime (24.8%) (Figure 2). Anti-MRSA therapy, when given, was administered for a median (IQR) of 3 (1-4) days while antipseudomonal β-lactam therapy was given for a median (IQR) of 4 (2-6) days.

### Total Fraction of Hospitalizations With Broad-Spectrum Therapy

**Figure 3.**
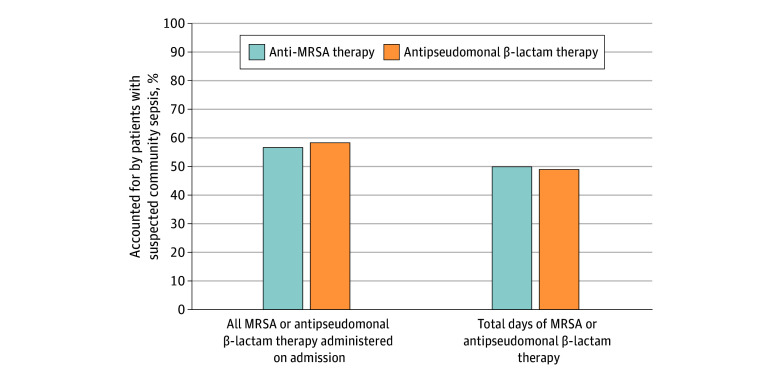
Fraction of Total Anti-MRSA and Antipseudomonal β-Lactam Therapy Accounted for by Patients With Suspected Community-Onset Sepsis MRSA indicates methicillin-resistant *Staphylococcus aureus*.

Across all admissions from the community during the study period, patients with suspected sepsis accounted for 379 987 of 666 109 patients (57.0%) who received anti-MRSA antibiotic therapy on admission and 513 811 of 875 907 patients (58.7%) who received antipseudomonal antibiotic therapy on admission (Figure 3). When considering total days of antibiotic therapy given across all hospitalizations (anytime), patients with suspected sepsis accounted for 1 573 673 of 3 141 300 anti-MRSA antibiotic days (50.1%) and 2 569 518 of 5 211 745 antipseudomonal β-lactam days (49.3%).

### Trends in Empiric Broad-Spectrum Antibiotic Use, Resistant Organisms, and Unnecessarily Broad vs Inappropriately Narrow Treatment

**Figure 4.**
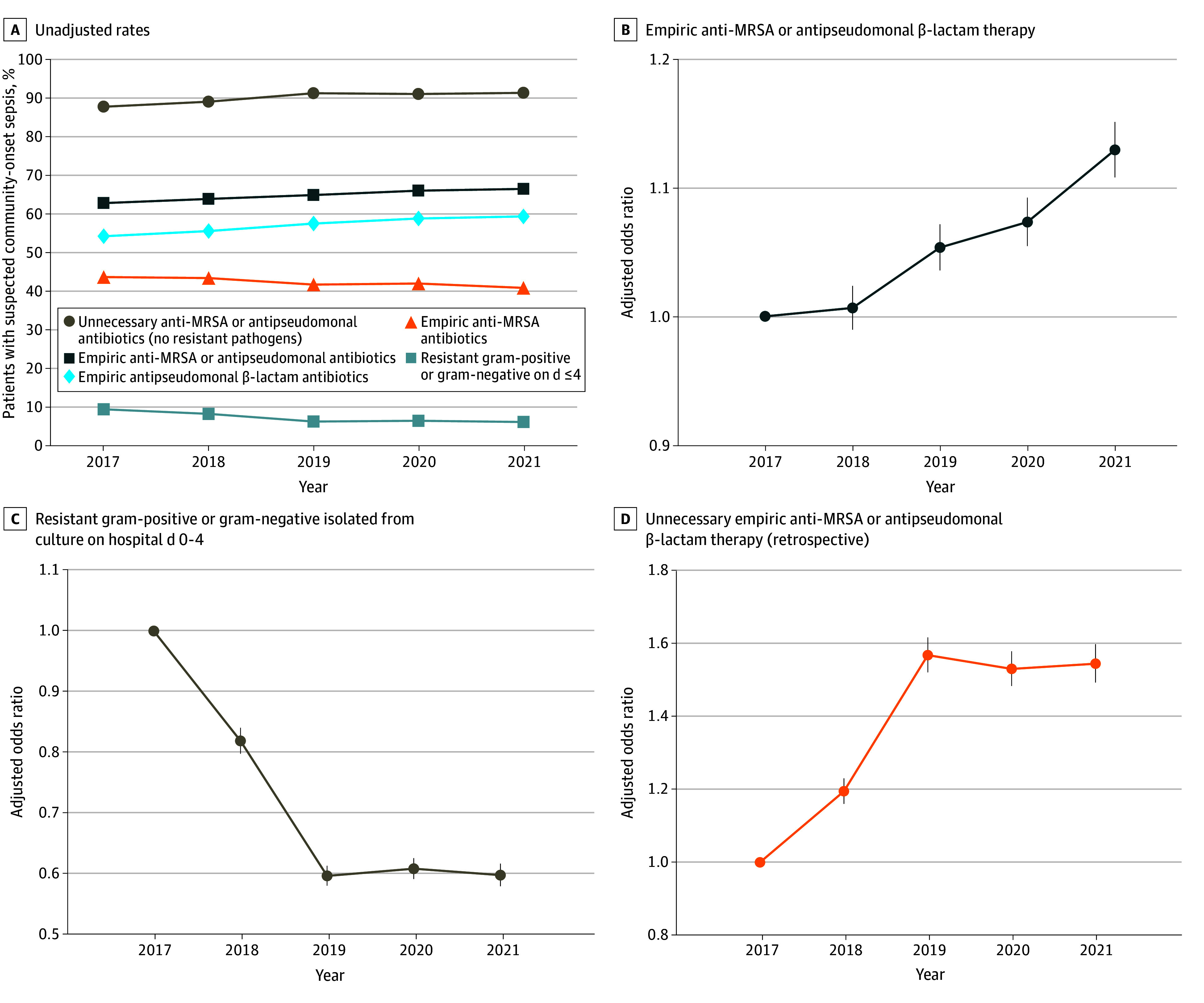
Trends in Empiric Broad-Spectrum Antibiotic Use for Community-Onset Suspected Sepsis MRSA methicillin-resistant *Staphylococcus aureus*.

Between 2017 and 2021, the annual fraction of patients with suspected community-onset sepsis treated with broad-spectrum therapy (either anti-MRSA or antipseudomonal therapy) increased from 63.0% (82 731 of 131 275 patients) in 2017 to 66.7% (101 003 of 151 435 patients) in 2021 (adjusted odds ratio [aOR] per year, 1.03; 95% CI, 1.03-1.04). This was driven by an increase in antipseudomonal β-lactam use, which increased from 54.4% in 2017 to 59.6% in 2021 (aOR per year, 1.07; 95% CI, 1.06-1.07); in contrast, anti-MRSA antibiotic use decreased from 43.9% to 41.0% (aOR per year, 0.95; 95% CI, 0.95-0.95) (Figure 4) (full model results and missing data rates shown in eTables 2 and 3 in Supplement 1).

The proportion of suspected sepsis episodes with resistant gram-positives on days 0 to 4 declined from 3.9% in 2017 to 3.0% in 2021 (aOR per year, 0.93; 95% CI, 0.92-0.94); episodes with resistant gram-negatives declined from 6.2% to 3.7% (aOR per year, 0.84; 95% CI, 0.83-0.85); and episodes with either a resistant gram-positive or gram-negative organism declined from 9.6% to 7.3% (aOR per year, 0.87; 95% CI, 0.87-0.88). This was driven by a decrease in the fraction of suspected sepsis patients who were culture-positive (32.8% in 2017 vs 19.8% in 2021; aOR per year, 0.81; 95% CI, 0.81-0.82); among culture-positive cases, there was relatively little change in the proportion with a resistant gram-positive organism (13.4% in 2017 vs 15.8% in 2021; aOR, 1.02; 95% CI, 1.01-1.04) or resistant gram-negative organism (18.8% in 2017 vs 18.2% in 2021; aOR, 0.95; 95% CI, 0.95-0.96) (eTable 4 in Supplement 1).

Among patients with suspected sepsis treated with empiric anti-MRSA therapy, no resistant gram-positive organisms were isolated in 356 311 of 379 987 cases (93.8%); this rate increased from 93.2% in 2017 to 94.2% in 2021 (aOR per year, 1.04; 95% CI, 1.03-1.05). Among patients treated with antipseudomonal β-lactams, no ceftriaxone-resistant gram-negative organism was isolated in 485 425 of 513 811 cases (94.5%); this fraction increased from 92.5% in 2017 to 95.1% in 2021 (aOR per year, 1.16; 95% CI, 1.14-1.17). Overall, among the 582 585 patients who received either empiric MRSA or antipseudomonal β-lactam therapy, 527 356 (90.5%) had neither resistant gram-positive nor gram-negative organisms isolated; this fraction increased from 88.0% in 2017 to 91.6% in 2021 (aOR per year, 1.12; 95% CI, 1.11-1.13) (Figure 4). Trends were similar in sensitivity analyses using broader and narrower definitions of suspected sepsis (eTable 5 in Supplement 1).

### Rates of Inappropriately Narrow Treatment for Resistant Pathogens

Among the 30 617 of 894 724 patients (3.4%) with suspected community-onset sepsis who had a resistant gram-positive organism isolated on hospital days 0 through 4, 6961 (22.7% of patients with resistant gram-positive infections, 0.8% of patients with suspected community-onset sepsis) did not receive empiric anti-MRSA therapy on admission. Among the 38 884 of 894 724 patients (4.3%) with resistant gram-negative infections, 10 458 (26.9% of patients with resistant gram-negative infections, 1.2% of patients with suspected community-onset sepsis) did not receive empiric antipseudomonal therapy. The fraction of patients who received inappropriately narrow treatment was stable over time for those with resistant gram-positive organisms (aOR per year, 0.98; 95% CI, 0.96-1.00) but decreased for patients with resistant gram-negative organisms (aOR per year, 0.89; 95% CI, 0.87-0.90) (eTable 6 in Supplement 1). For comparison, the number of patients with suspected sepsis who received anti-MRSA therapy but did not have resistant gram-positive organisms was 356 331 (51.2 times more than the number of resistant gram-positive cases that were undertreated [6961 cases]), and the number who received antipseudomonal therapy but did not have resistant gram-negative organisms was 485 425 (46.4 times more than the number of resistant gram-negative cases that were undertreated [10 458 cases]).

### Trends in In-Hospital Death and Hospice Discharge

Overall, 40 124 patients with suspected community-onset sepsis (4.5%) died in-hospital while another 41 953 (4.7%) were discharged to hospice. The combined outcome of in-hospital death or discharge to hospice was similar in 2017 (8.7%), 2018 (8.6%), and 2019 (8.6%) but increased during the COVID-19 pandemic in 2020 (10.0%) and 2021 (10.1%).

## Discussion

Among 6.3 million adults admitted to 241 US hospitals between 2017 and 2021, half of all anti-MRSA and antipseudomonal β-lactam prescribing was for suspected community-onset sepsis but only 7% of patients with community-onset sepsis overall, and 9.5% of patients treated with broad-spectrum agents, had positive cultures for MRSA, *Pseudomonas aeruginosa*, or other resistant organisms early in their hospitalization. Broad-spectrum antibiotic use for patients with suspected community-onset sepsis rose over time but the percentage of cases with resistant organisms decreased. There was a decrease in patients receiving inappropriately narrow therapy but the absolute number who received unnecessarily broad treatment was nearly 50-fold higher than the number who were undertreated. The increase in broad-spectrum antibiotic prescribing was not associated with improvements in mortality for patients with suspected community-onset sepsis during the study period.

Others have also documented that MRSA and ceftriaxone-resistant gram-negative organisms constitute a small minority of culture-positive community-onset sepsis cases.^14,30^ Our study extends these investigations by evaluating trends over time, quantifying the contribution of empiric broad-spectrum prescribing to overall antibiotic use in hospitals, and adding data about patients with culture-negative sepsis and those with clinically suspected but unconfirmed sepsis. Including these patients is critical to provide a comprehensive picture of antibiotic use for suspected sepsis because approximately one-third of patients initially treated for possible sepsis end up having noninfectious diagnoses and culture-positive sepsis accounts for a minority of cases (only 24% in our study).^12,13,31,32,33^ Documenting the large contribution of suspected sepsis to overall broad-spectrum antibiotic utilization underscores the importance of efforts to steward empiric antibiotic therapy in this population for whom broad-spectrum antibiotic prescribing has been strongly encouraged.

There are several possible reasons why broad-spectrum antibiotic use for suspected sepsis is increasing, including the ongoing success of the Surviving Sepsis Campaign, the implementation of SEP-1, state sepsis regulatory measures, and the sepsis quality improvement initiatives these measures have catalyzed. This appears to have counterbalanced the contemporaneous increase in antibiotic stewardship programs in the US, which tend to focus more on deescalating antibiotics rather than narrowing empiric treatment regimens for suspected sepsis.^34^

The finding that a decreasing proportion of patients with suspected community-onset sepsis had resistant pathogens appears paradoxical given that broad-spectrum antibiotic use increased in this population over time. However, this trend was driven primarily by decreasing rates of culture positivity among patients with suspected community-onset sepsis whereas there was minimal change in the proportion of culture-positive patients with resistant organisms. The decrease in culture-positive cases may reflect an increasing tendency to suspect and treat patients for sepsis who turn out not to have sepsis in retrospect. Alternatively, it is possible that community-onset sepsis cases caused by resistant pathogens have truly decreased; indeed, other studies have reported recent declines in community-onset MRSA, *Pseudomonas*, and *Acinetobacter* infections.^35,36,37^ This may reflect the fitness cost of resistance, antibiotic stewardship efforts in both inpatient and outpatient settings, greater attention to hospital infection prevention programs, and changing antibiotic use practices in the environment and in animals.^38^ Notably, increases in multidrug-resistant organisms were observed in US hospitals during the early COVID-19 pandemic era; however, these were primarily driven by hospital-onset rather than community-onset infections.^39^

The increasing mismatch between empiric prescribing and identified pathogens constitutes a patient-safety risk. Prior investigations suggest that overly broad antibiotics are associated with similarly poor outcomes as inappropriately narrow antibiotics.^14,40,41^ Among 17 430 adults treated for community-onset sepsis in 104 hospitals, for example, both overly broad and overly narrow antibiotics were associated with a 20% increase in the odds of hospital death; overly broad regimens were further associated with increased risk of acute kidney injury and *Clostridioides difficile* infections.^14^

We stress that our results should not be interpreted as critical of prescription standards of front-line providers, nor should our description of certain antibiotics courses as unnecessary in retrospect be taken to mean inappropriate at the time of prescribing, because in many cases they were guideline compliant. Additionally, front-line clinicians often must make treatment decisions with incomplete information. This leads many to err on the side of broader coverage, particularly when patients are severely ill. Our analysis was also not designed to identify clinical factors or algorithms that clinicians can use to determine which patients can safely tolerate narrow-spectrum antibiotics and which require broad-spectrum treatment. Our study was rather designed to gather epidemiologic data to inform guideline and policy deliberations around finding the balance between minimizing undertreatment of patients with serious infections vs minimizing overuse of antibiotic at both the individual and population levels. Several professional societies, including the Infectious Diseases Society of America, have recently encouraged regulators and guideline developers to provide more specific direction about which patients with possible sepsis require immediate broad-spectrum antibiotics, which can safely be managed with narrower spectrum regimens, and which can be managed without antibiotics while obtaining further data to clarify their diagnoses.^15,18^

### Limitations

Our study has several limitations. First, some antibiotic-resistant infections may have been missed because cultures were not obtained (eg, sputum for pneumonia or sampling from intra-abdominal infections). Some cultures may also have been falsely negative due to prior antibiotic administration. However, our findings were consistent in a sensitivity analysis restricted to patients with positive cultures. Conversely, some positive cultures may have represented colonization rather than invasive infection. However, the findings reflect real clinical scenarios where colonization vs infection is often difficult to discern. Second, we inferred suspicion for sepsis based on clinical actions, namely blood culture orders, lactate measurements, and intravenous antibiotics; this definition may overestimate or underestimate the true incidence of suspected sepsis. We took this approach because documentation of suspected sepsis is subjective, variable, and tends to be biased toward patients with severe illness and overt infection.^42,43^ However, we conducted sensitivity analyses using broader and narrower definitions of suspected sepsis and found similar trends. Third, we focused on community-onset sepsis rather than hospital-onset sepsis (where resistant organisms are more prevalent), thus precluding generalizing our findings to all patients with sepsis. We also excluded patients admitted from health care facilities who tend to have higher rates of antibiotic exposure and drug-resistant organisms.^44,45^ Nonetheless, community-onset sepsis accounts for the majority of sepsis cases.^46^ Fourth, we were unable to characterize recent health care exposures because they could not reliably be determined in our dataset. Fifth, we did not account for allergies as a potential reason for broad-spectrum antibiotic use (eg, vancomycin in a patient with a β-lactam allergy), nor the neutropenic population where guidelines recommend antipseudomonal coverage even when cultures are negative. Sixth, we included VRE in our definition of resistant gram-positive organisms, but ampicillin-resistant enterococci could also be considered a pathogen of interest given that vancomycin (rather than daptomycin or linezolid) was the primary anti-MRSA antibiotic administered in our cohort.

## Conclusions

Empiric treatment for suspected community-onset sepsis accounts for about half of broad-spectrum antibiotic use in US hospitals even though resistant organisms are isolated in only 7% of patients with community-onset sepsis and less than 10% of patients treated with broad-spectrum agents. Broad-spectrum antibiotic use for suspected sepsis is increasing while the percentage of cases with proven resistant infections is decreasing, translating into an increasing number of broad-spectrum courses that are likely unnecessary in retrospect. These findings help elucidate the contribution of suspected sepsis to antibiotic prescribing in US hospitals and inform ongoing deliberations on finding the balance between assuring early, appropriate therapy for patients with severe infections vs limiting overuse for the majority of patients who do not have antibiotic-resistant infections.

## Notes

Supplement 1.
eMethods.
**eFigure.** Study Cohort Flowchart **eTable 1.** Characteristics of Study Hospitals **eTable 2.** Full Adjusted Model Results for Empiric Broad-Spectrum Antibiotic Use (Anti-MRSA, Antipseudomonal Beta-Lactam Therapy, or Either) for Suspected Community-Onset Sepsis **eTable 3.** Rates of Missing Data for Laboratory Values Used in Models **eTable 4.** Proportion of Suspected Community-Onset Sepsis Cases with Positive Cultures on Days 0-4 and Proportion of Culture-Positive Cases with Resistant Gram-Positive or Resistant Gram-Negative Organisms **eTable 5.** Sensitivity Analyses: Unadjusted Rates and Adjusted Trends for Broader and Narrower Definitions of Suspected Sepsis and Among Culture-Positive Patients **eTable 6.** Rates of Inappropriately Narrow Empiric Treatment for Patients with Suspected Sepsis with Resistant Gram-Positive or Gram-Negative Pathogens

Supplement 2.
Data Sharing Statement
