# Supplementary material for: Trends in Empiric Broad-Spectrum Antibiotic Use for Suspected Community-Onset Sepsis in US Hospitals
Source: JAMA Netw Open. 2024 Jun 27;7(6):e2418923. doi: 10.1001/jamanetworkopen.2024.18923 (PMC11211962; doi:10.1001/jamanetworkopen.2024.18923)
Supplement: Supplement 1. — eMethods. eFigure. Study Cohort Flowchart eTable 1. Characteristics of Study Hospitals eTable 2. Full Adjusted Model Results for Empiric Broad-Spectrum Antibiotic Use (Anti-MRSA, Antipseudomonal Beta-Lactam Therapy, or Either) for Suspected Community-Onset Sepsis eTable 3. Rates of Missing Data for Laboratory Values Used in Models eTable 4. Proportion of Suspected Community-Onset Sepsis Cases with Positive Cultures on Days 0-4 and Proportion of Culture-Positive Cases with Resistant Gram-Positive or Resistant Gram-Negative Organisms eTable 5. Sensitivity Analyses: Unadjusted Rates and Adjusted Trends for Broader and Narrower Definitions of Suspected Sepsis and Among Culture-Positive Patients eTable 6. Rates of Inappropriately Narrow Empiric Treatment for Patients with Suspected Sepsis with Resistant Gram-Positive or Gram-Negative Pathogens [file jamanetwopen-e2418923-s001.pdf]

Supplemental Online Content

Rhee C, Chen T, Kadri SS, et al; CDC Prevention Epicenters Program. Trends in empiric broad-spectrum antibiotic use for suspected community-onset sepsis in US hospitals. *JAMA Netw Open.* 2024;7(6):e2418923. doi:10.1001/jamanetworkopen.2024.18923

eMethods.

eFigure. Study Cohort Flowchart

eTable 1. Characteristics of Study Hospitals

eTable 2. Full Adjusted Model Results for Empiric Broad-Spectrum Antibiotic Use (Anti-MRSA, Antipseudomonal Beta-Lactam Therapy, or Either) for Suspected Community-Onset Sepsis

eTable 3. Rates of Missing Data for Laboratory Values Used in Models

eTable 4. Proportion of Suspected Community-Onset Sepsis Cases with Positive Cultures on Days 0-4 and Proportion of Culture-Positive Cases with Resistant Gram-Positive or Resistant Gram-Negative Organisms

eTable 5. Sensitivity Analyses: Unadjusted Rates and Adjusted Trends for Broader and Narrower Definitions of Suspected Sepsis and Among Culture-Positive Patients

eTable 6. Rates of Inappropriately Narrow Empiric Treatment for Patients with Suspected Sepsis with Resistant Gram-Positive or Gram-Negative Pathogens

This supplemental material has been provided by the authors to give readers additional information about their work.

© 2024 Rhee C et al. *JAMA Network Open.*

eMethods

A. Definitions of Anti-MRSA and Antipseudomonal Beta-Lactam Antibiotics

Anti-MRSA antibiotics included intravenous vancomycin, daptomycin, linezolid (intravenous or oral), and ceftaroline.

Antipseudomonal beta-lactam antibiotics included ceftazidime, cefepime, piperacillin-tazobactam, aztreonam, imipenem, meropenem, doripenem, ceftazidime-avibactam, ceftolozane-tazobactam, imipenem-relebactam, meropenem-vaborbactam, and cefiderocol.

B. Definitions of Anti-MRSA and Antipseudomonal Beta-Lactam Antibiotics

Resistant Gram-Positive Organisms

1. **Methicillin-resistant *Staphylococcus aureus* (MRSA)** = *S.aureus* resistant to ≥1 of the following: methicillin, oxacillin, cefazolin, or cefotixin
2. **Vancomycin-resistant enterococcus (VRE)** = any Enterococcus species resistant to vancomycin
3. **Ceftriaxone-resistant Streptococcus** = any Streptococcus species with the following resistance pattern:
    a. Resistant to ceftriaxone OR cefotaxime, OR
    b. No susceptibility reported to ceftriaxone or cefotaxime, but resistant to penicillin
4. **Methicillin-resistant coagulase negative Staphylococcus bacteremia** = coagulase-negative staphylococcus resistant to ≥1 of the following: methicillin, oxacillin, cefazolin, or cefotixin, isolated from separate blood cultures with different specimen IDs on the same or consecutive calendar days.

Ceftriaxone-Resistant Gram-Negative Organisms

1. Pseudomonas aeruginosa (any)
2. Enterobacterales resistant to ceftriaxone OR cefotaxime or higher generation cephalosporin
3. Non-fermenting gram-negative rod (other than Pseudomona aeruginosa), resistant to ceftriaxone or cefotaxime or higher generation cephalosporin
4. Any other gram-negative organism resistant to ceftriaxone or cefotaxime or higher generation cephalosporin
5. AmpC-producing Enterobacterales - one of the following: *Enterobacter cloacae*, *Klebsiella aerogenes*, or *Citrobacter freundii*, regardless of ceftriaxone susceptibility
6. *Stenotrophomonas maltophilia*
7. *Achromobacter* species
8. *Burkholderia* species

© 2024 Rhee C et al. *JAMA Network Open.*

eFigure. Study Cohort Flowchart

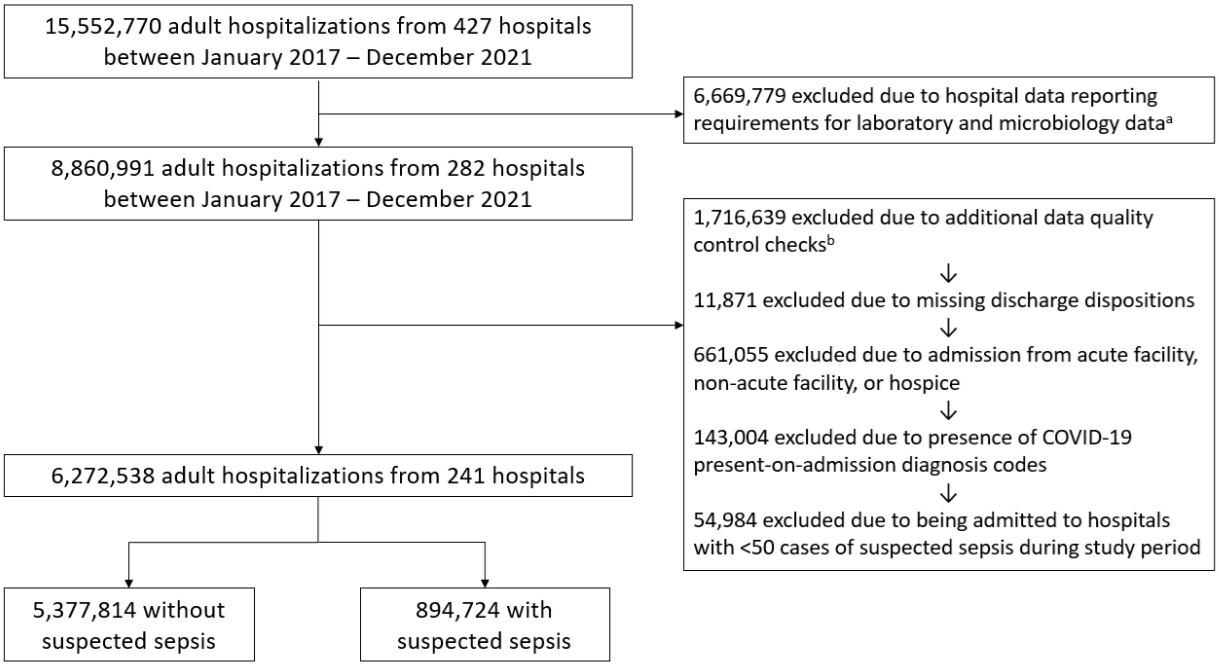

[a] In this initial data quality control stage, exclusion criteria included:

- Encounters from hospital-months that did not report any microbiology data
- Encounters from hospital-months that did not report any laboratory data or where <70% of encounters had a creatinine and platelet measurement

[b] In this second data quality control stage, exclusion criteria included:

- Encounters from hospital-months with <50 encounters
- Encounters from hospital-months where the number of encounters was <25% of the median monthly number of encounters for that hospital during the entire study period (likely indicating a lapse in data reporting)
- Encounters from hospital-months where <90% of encounters have a medication administered
- Encounters from hospital-months where the blood culture sampling rate was <25% of the median of rate of blood culture sampling in other months (indicating a data quality issue with blood culture reporting)
- Encounters from hospital-months where <75% of encounters with length-of-stay of ≥5 days or with an ICU stay had non-missing creatinine and platelet values (indicating a lab data quality issue)
- Encounters from hospital-months with no blood cultures or where <50% of patients with ICD-10 sepsis codes had a blood culture drawn (indicating a data quality issue with blood culture reporting)
- Encounters from hospital-months in which >75% of encounters with positive *S.aureus*, Enterococcus, or gram-negative pathogens from blood cultures, lower respiratory, or genitourinary cultures have antibiotic susceptibility data reported (to ensure the cohort only included hospitals that reported antimicrobial susceptibility data)
- Patients in the ED for >1 day before admission – this was necessary to anchor our analyses to a short, defined time period following patients' presentation to the hospital

© 2024 Rhee C et al. *JAMA Network Open.*

eTable 1. Characteristics of Study Hospitals

Hospital Characteristic	Study Hospitals (Total N=241)
**Bed Size**	
Small (<200 beds)	125 (51.9%)
Medium (200-499 beds)	87 (36.1%)
Large (500+ beds)	29 (12.0%)
**Teaching Status**	
Teaching Hospital	68 (28.2%)
Non-Teaching Hospital	173 (71.8%)
**Urbanicity**	
Urban	169 (70.1%)
Rural	72 (29.9%)
**Region**	
Northeast	34 (14.1%)
Midwest	53 (22.0%)
South	137 (56.9%)
West	17 (7.1%)

113/241 (46.9%) hospitals reported data in each year during the study period.

© 2024 Rhee C et al. *JAMA Network Open.*

eTable 2. Full Adjusted Model Results for Empiric Broad-Spectrum Antibiotic Use (Anti-MRSA, Antipseudomonal Beta-Lactam Therapy, or Either) for Suspected Community-Onset Sepsis

The mixed-effects logistic regression model shown here uses the primary definition for suspected community-onset sepsis (blood culture drawn, lactate measured, and intravenous antibiotic all on hospital day ≤1). Physiologic variables (laboratory values, vasopressors, ventilatory support) used the worst value on hospital day 0, 1, or 2. Similar models were used for all other adjusted analyses.

	**Anti-MRSA Therapy**		**Antipseudomonal Beta-Lactam Therapy**		**Anti-MRSA OR Antipseudomonal Beta-Lactam Therapy**	
Predictors	***Odds Ratios (95% CI)***	***p-value***	***Odds Ratios (95% CI)***	***p-value***	***Odds Ratios (95% CI)***	***p-value***
Admission year	0.95 (0.95 – 0.95)	**<0.001**	1.06 (1.06 – 1.07)	**<0.001**	1.03 (1.03 – 1.04)	**<0.001**
urban or rural hospital [ref=URBAN]	1.09 (0.95 – 1.24)	0.225	1.16 (0.99 – 1.35)	0.062	1.17 (1.01 – 1.34)	**0.030**
Teaching hospital [ref=YES]	1.26 (1.09 – 1.46)	**0.002**	1.04 (0.88 – 1.23)	0.658	1.06 (0.91 – 1.23)	0.469
hospital bed size (medium – 200-499 beds) [ref=large – 500+ beds]	0.84 (0.68 – 1.02)	0.084	0.74 (0.58 – 0.94)	**0.012**	0.74 (0.60 – 0.91)	**0.005**
hospital bed size [small - <200 beds]	0.66 (0.53 – 0.81)	**<0.001**	0.62 (0.49 – 0.80)	**<0.001**	0.61 (0.49 – 0.75)	**<0.001**
hospital region (NORTHEAST [ref=Midwest]	1.00 (0.82 – 1.21)	0.961	0.85 (0.68 – 1.07)	0.166	0.93 (0.76 – 1.14)	0.479
hospital region (SOUTH)	0.95 (0.82 – 1.10)	0.513	1.24 (1.04 – 1.46)	**0.014**	1.22 (1.05 – 1.42)	**0.011**
hospital region (WEST)	1.01 (0.79 – 1.29)	0.961	0.63 (0.47 – 0.84)	**0.002**	0.79 (0.60 – 1.02)	0.076
Age (per 1 year increase)	0.99 (0.99 – 0.99)	**<0.001**	1.00 (1.00 – 1.00)	**<0.001**	0.99 (0.99 – 0.99)	**<0.001**
Male sex	1.30 (1.29 – 1.31)	**<0.001**	1.29 (1.28 – 1.30)	**<0.001**	1.33 (1.32 – 1.34)	**<0.001**
race ethnicity cat (black) [ref=white]	1.03 (1.01 – 1.04)	**0.002**	1.03 (1.01 – 1.04)	**0.002**	0.98 (0.96 – 1.00)	**0.017**
race ethnicity cat (Asian)	0.91 (0.87 – 0.94)	**<0.001**	1.12 (1.08 – 1.16)	**<0.001**	1.08 (0.97 – 1.05)	0.700
race ethnicity cat (Other)	1.02 (0.99 – 1.05)	0.128	1.06 (1.03 – 1.09)	**<0.001**	1.03 (1.00 – 1.06)	**0.048**

© 2024 Rhee C et al. *JAMA Network Open.*

race ethnicity cat (Unknown)	0.98 (0.93 – 1.04)	0.518	1.00 (0.94 – 1.06)	0.982	1.00 (0.94 – 1.06)	0.996
race ethnicity cat (Hispanic)	0.941 (0.92 – 0.96)	**<0.001**	1.00 (0.98 – 1.02)	0.715	0.95 (0.93 – 0.98)	**<0.001**
lactate ≤2.0 [ref=missing]	1.65 (1.62 – 1.67)	**<0.001**	1.84 (1.82 – 1.86)	**<0.001**	1.74 (1.72 – 1.76)	**<0.001**
lactate 2.0-3.9	2.12 (2.09 – 2.15)	**<0.001**	2.44 (2.41 – 2.47)	**<0.001**	2.30 (2.27 – 2.33)	**<0.001**
lactate ≥4.0	2.81 (2.76 – 2.86)	**<0.001**	3.37 (3.31 – 3.44)	**<0.001**	3.18 (3.12 – 3.24)	**<0.001**
creatinine 1.2 to <2.0 [ref=<1.2 or missing)	0.95 (0.94 – 0.96)	**<0.001**	0.97 (0.96 – 0.98)	**<0.001**	0.95 (0.94 – 0.96)	**<0.001**
creatinine 2.0 to <3.5	1.04 (1.02 – 1.06)	**<0.001**	1.08 (1.07 – 1.10)	**<0.001**	1.07 (1.05 – 1.09)	**<0.001**
creatinine 3.5 to <5.0	1.23 (1.20 – 1.27)	**<0.001**	1.25 (1.22 – 1.29)	**<0.001**	1.29 (1.25 – 1.33)	**<0.001**
creatinine ≥5.0	1.52 (1.49 – 1.56)	**<0.001**	1.35 (1.31 – 1.38)	**<0.001**	1.50 (1.46 – 1.55)	**<0.001**
platelets 100-149 [ref=≥150 or missing]	1.06 (1.04 – 1.08)	**<0.001**	1.00 (0.98 – 1.02)	0.895	1.01 (0.99 – 1.03)	0.325
platelets 50-99	1.16 (1.14 – 1.19)	**<0.001**	1.07 (1.04 – 1.10)	**<0.001**	1.08 (1.05 – 1.11)	**<0.001**
platelets 20-49	1.43 (1.36 – 1.49)	**<0.001**	1.42 (1.35 – 1.49)	**<0.001**	1.46 (1.38 – 1.55)	**<0.001**
platelets <20	1.80 (1.67 – 1.94)	**<0.001**	2.92 (2.64 – 3.24)	**<0.001**	3.03 (2.70 – 3.40)	**<0.001**
wbc <4 [ref=4-12]	1.67 (1.62 – 1.71)	**<0.001**	2.04 (1.98 – 2.10)	**<0.001**	2.00 (1.94 – 2.06)	**<0.001**
wbc 12 to 20	1.14 (1.13 – 1.15)	**<0.001**	1.26 (1.24 – 1.27)	**<0.001**	1.24 (1.22 – 1.25)	**<0.001**
wbc >20	1.61 (1.59 – 1.63)	**<0.001**	1.80 (1.77 – 1.83)	**<0.001**	1.83 (1.80 – 1.86)	**<0.001**
bilirubin 1.2 to <2.0 [ref=<1.2 or missing]	0.92 (0.90 – 0.93)	**<0.001**	0.99 (0.98 – 1.01)	0.353	0.97 (0.95 – 0.98)	**<0.001**
bilirubin 2.0 to <6.0	0.83 (0.81 – 0.85)	**<0.001**	1.06 (1.03 – 1.08)	**<0.001**	1.01 (0.985 – 1.04)	0.421
bilirubin 6.0 to <12.0	0.68 (0.64 – 0.72)	**<0.001**	1.04 (0.98 – 1.11)	0.168	0.94 (0.88 – 1.00)	**0.048**
bilirubin ≥12.0	0.48 (0.44 – 0.52)	**<0.001**	0.79 (0.73 – 0.86)	**<0.001**	0.66 (0.61 – 0.72)	**<0.001**

© 2024 Rhee C et al. *JAMA Network Open.*

max vent support (non-invasive ventilation)	1.04 (1.02 – 1.06)	**<0.001**	1.03 (1.01 – 1.05)	**0.006**	1.00 (0.98 – 1.02)	0.803
max vent support (invasive ventilation)	1.24 (1.21 – 1.26)	**<0.001**	1.30 (1.27 – 1.33)	**<0.001**	1.37 (1.33 – 1.40)	**<0.001**
Vasopressor administration	1.90 (1.86 – 1.93)	**<0.001**	1.99 (1.95 – 2.03)	**<0.001**	2.19 (2.14 – 2.24)	**<0.001**
Intra-abdominal infection diagnosis POA [ref=Pulmonary infection]	0.54 (0.53 – 0.55)	**<0.001**	2.08 (2.04 – 2.12)	**<0.001**	2.09 (2.05 – 2.13)	**<0.001**
Genitourinary infection diagnosis POA	0.60 (0.59 – 0.61)	**<0.001**	0.92 (0.91 – 0.94)	**<0.001**	0.95 (0.94 – 0.97)	**<0.001**
Skin/soft tissue infection diagnosis POA	8.13 (7.99 – 8.29)	**<0.001**	2.03 (2.00 – 2.07)	**<0.001**	8.11 (7.93 – 8.29)	**<0.001**
None of the above infection diagnoses POA	1.05 (1.034 – 1.06)	**<0.001**	1.20 (1.18 – 1.21)	**<0.001**	1.49 (1.47 – 1.51)	**<0.001**
AHRQ Elixhauser mortality index (per 1 point increase)	1.08 (1.08 – 1.09)	**<0.001**	1.08 (1.07 – 1.08)	**<0.001**	1.09 (1.08 – 1.09)	**<0.001**

© 2024 Rhee C et al. *JAMA Network Open.*

eTable 3. Rates of Missing Data for Laboratory Values

This table shows the number of patients with suspected community-onset sepsis (using the primary definition) that had missing values for the laboratory values used in the mixed-effects logistic regression models on hospital days 0, 1, or 2. By definition, the rate of missingness for lactate measurements was zero.

Laboratory Parameter	No. Missing (N = 894,724 for Suspected Community-Onset Sepsis
Creatinine	650 (0.07%)
Platelets	1,066 (0.1%)
White blood cell count	1,100 (0.1%)
Bilirubin	85,447 (9.6%)

© 2024 Rhee C et al. *JAMA Network Open.*

eTable 4. Proportion of Suspected Community-Onset Sepsis Cases with Positive Cultures on Days 0-4 and Proportion of Culture-Positive Cases with Resistant Gram-Positive or Resistant Gram-Negative Organisms

Outcome	2017	2018	2019	2020	2021	Adjusted OR/year [95% CI]
Proportion of Suspected Community-Onset Sepsis Cases with Positive Cultures on Day 0-4	43,114 / 131,275 (32.8%)	51,946 / 192,413 (27.0%)	43,996 / 217,596 (20.2%)	41,650 / 202,005 (20.6%)	30,022 / 151,435 (19.8%)	0.81 [0.81-0.82]
Proportion of Culture-Positive Sepsis Cases with Resistant Gram-Positive Organism	5,126 / 43,114 (13.4%)	7,270 / 51,946 (14.0%)	7,019 / 43,996 (16.0%)	6,593 / 41,650 (15.8%)	4,609 / 30,002 (15.4%)	1.02 [1.01-1.04]
Proportion of Culture-Positive Sepsis Cases with Resistant Gram-Negative Organism	8,101 / 43,114 (18.8%)	9,834 / 51,946 (18.9%)	7,800 / 43,996 (17.7%)	7,572 / 41,650 (18.2%)	5,537 / 30,002 (18.4%)	0.95 [0.95-0.96]

© 2024 Rhee C et al. *JAMA Network Open.*

eTable 5. Sensitivity Analyses: Unadjusted Rates and Adjusted Trends for Broader and Narrower Definitions of Suspected Sepsis and Among Culture-Positive Patients

Outcome	2017	2018	2019	2020	2021	Adjusted OR/year [95% CI]
**Primary Definition of Suspected Sepsis (Blood Culture, IV Antibiotic, and Lactate Measurement)**						
Empiric Anti-MRSA or PsA Antibiotic Use	82,731 / 131,275 (63.0%)	123,352 / 192,413 (64.1%)	141,678 / 217,596 (65.1%)	133,821 / 202,005 (66.2%)	101,003 / 151,435 (66.7%)	1.03 [1.03-1.04]
Resistant Gram Positive or Gram Negative Organism on Day ≤4	12,567 / 131,275 (9.6%)	16,121 / 192,413 (8.4%)	13,932 / 217,596 (6.4%)	13,307 / 202,005 (6.6%)	9,507 / 151,435 (6.3%)	0.87 [0.87-0.88]
Unnecessarily Broad Anti-MRSA or PsA Antibiotics	72,793 / 82,731 (88.0%)	110,170 / 123,352 (89.3%)	129,635 / 141,678 (91.5%)	122,194 / 133,821 (91.3%)	92,564 / 101,003 (91.6%)	1.12 [1.11-1.13]
**Broader Definition of Suspected Sepsis (Blood Culture Only)**						
Empiric Anti-MRSA or PsA Antibiotic Use	102,570 / 210,951 (48.6%)	147,318 / 290,993 (50.6%)	164,539 / 317,851 (23.9%)	153,227 / 290,910 (52.7%)	115,528 / 218,135 (53.0%)	1.034 [1.03-1.31]
Resistant Gram Positive or Gram Negative Organism on Day ≤4	17,043 / 210,951 (8.1%)	21,256 / 290,993 (7.3%)	17,801 / 317,851 (5.6%)	16,571 / 290,910 (5.7%)	11,908 / 218,135 (5.5%)	0.88 [0.87-0.88]
Unnecessarily Broad Anti-MRSA or PsA Antibiotics	90,219 / 102,570 (88.0%)	131,297 / 147,318 (89.1%)	150,184 / 164,539 (91.3%)	139,619 / 153,227 (91.1%)	105,674 / 115,528 (91.5%)	1.05 [1.05-1.06]
**Narrower Definition of Suspected Sepsis (Requires Organ Dysfunction*)**						
Empiric MRSA or PsA Antibiotic Use	45,473 / 66,262 (68.6%)	67,859 / 98,024 (69.2%)	77,643 / 111,206 (69.8%)	74,722 / 106,190 (70.4%)	55,670 / 78,967 (70.5%)	1.03 [1.02-1.03]
Resistant Gram Positive or Gram Negative Organism on Day ≤4	6,392 / 66,262 (9.7%)	8,133 / 98,024 (8.3%)	6,991 / 111,206 (6.3%)	6,962 / 106,190 (6.6%)	4,798 / 78,967 (6.1%)	0.87 [0.86-0.88]
Unnecessarily Broad Anti-MRSA or PsA Antibiotics	40,234 / 45,473 (88.5%)	61,051 / 67,859 (90.0%)	71,547 / 77,643 (92.2%)	68,614 / 74,722 (91.8%)	51,381 / 55,670 (92.3%)	1.13 [1.12-1.14]
**Culture-Positive Sepsis (Using Primary Definition of Suspected Sepsis)**						
Empiric Anti-MRSA or PsA Antibiotic Use	29,906 / 43,114 (69.4%)	38,124 / 51,946 (73.4%)	35,798 / 43,996 (81.4%)	34,169 / 41,650 (82.0%)	24,727 / 30,022 (82.4%)	1.11 [1.10-1.12]
Resistant Gram Positive or Gram Negative Organism On Day ≤4	11,570 / 43,114 (26.8%)	14,756 / 51,946 (28.4%)	12,361 / 43,996 (28.1%)	11,837 / 41,650 (28.4%)	8,451 / 30,022 (28.1%)	0.98 [0.97-0.99]
Unnecessarily Broad Anti-MRSA or PsA Antibiotics	24,65 / 35,991 (68.5%)	30,548 / 45,231 (23.8%)	28,723 / 41,554 (21.9%)	26,794 / 39,031 (20.5%)	19,410 / 28,240 (68.7%)	1.03 [1.02-1.03]

*Organ dysfunction was adapted from the Centers for Disease Control and Prevention's Adult Sepsis Event "eSOFA" criteria. Criteria included any of the following on hospital day 0 or 1:

- Systolic blood pressure <90 mmHg or vasopressor
- Non-invasive ventilation or invasive mechanical ventilation
- Lactate >2.0 mmol/L

© 2024 Rhee C et al. *JAMA Network Open.*

- Creatinine >2.0, excluding patients with Elixhauser diagnoses of Severe Renal Failure that was Present on Admission
- Bilirubin >2.0, excluding patients with Elixhauser diagnoses of Severe Liver Disease that was Present on Admission
- Platelets <100, excluding patients with Elixhauser diagnoses of Leukemia, Lymphoma, Metastatic Cancer, or Malignant Solid Tumor without Metastasis

© 2024 Rhee C et al. *JAMA Network Open.*

eTable 6. Rates of Inappropriately Narrow Empiric Treatment for Patients with Suspected Sepsis with Resistant Gram-Positive or Gram-Negative Pathogens

Outcome	2017	2018	2019	2020	2021	Adjusted OR/year [95% CI]
**Suspected Sepsis with Resistant Gram-Positive Pathogens (n=30,617)**						
**No Empiric Anti-MRSA Antibiotic Use**	1,226 / 5,126 (23.9%)	1,789 / 7,270 (24.6%)	1,596 / 7,019 (22.7%)	1,367 / 6,593 (20.7%)	983 / 4,609 (21.3%)	0.98 [0.96-1.00]
**Suspected Sepsis with Resistant Gram-Negative Pathogens (n=38,844)**						
**No Empiric Anti-PsA Antibiotic Use**	2,708 / 8,108 (33.4%)	2,975 / 9,834 (30.3%)	1,853 / 7,800 (238%)	1,769 / 7,572 (23.4%)	1,153 / 5,537 (20.8%)	0.89 [0.87-0.90]

© 2024 Rhee C et al. *JAMA Network Open.*
