# Supplementary material for: Trends in Empiric Broad-Spectrum Antibiotic Use for Suspected Community-Onset Sepsis in US Hospitals
Source: JAMA Netw Open. 2024 Jun 27;7(6):e2418923. doi: 10.1001/jamanetworkopen.2024.18923 (PMC11211962; doi:10.1001/jamanetworkopen.2024.18923)
Supplement: Supplement 2. — Data Sharing Statement [file jamanetwopen-e2418923-s002.pdf]

Data Sharing Statement

Rhee. Trends in Empiric Broad-Spectrum Antibiotic Use for Suspected Community-Onset Sepsis in US Hospitals. *JAMA Netw Open*. Published June 27, 2024.
doi:10.1001/jamanetworkopen.2024.18923

Data
Data available: No
